# Green Composites Made of Bamboo Fabric and Poly (Lactic) Acid for Packaging Applications—A Review

**DOI:** 10.3390/ma9060435

**Published:** 2016-06-01

**Authors:** M.R. Nurul Fazita, Krishnan Jayaraman, Debes Bhattacharyya, M.K. Mohamad Haafiz, Chaturbhuj K. Saurabh, M. Hazwan Hussin, Abdul Khalil H.P.S.

**Affiliations:** 1 Centre for Advanced Composite Materials (CACM), Department of Mechanical Engineering, University of Auckland, Private Bag 92019, Auckland 1142, New Zealand; 2 School of Industrial Technology, Universiti Sains Malaysia, 11800 Gelugor, Penang, Malaysia; 3 Cluster for Polymer Composites, Science and Engineering Research Center, University Sains Malaysia, 14300 Nibong Tebal, Penang, Malaysia; 4 School of Chemical Sciences, Universiti Sains Malaysia, 11800 Gelugor, Penang, Malaysia

**Keywords:** natural fibre, woven fabric, bamboo fabric, biopolymer, poly (lactic) acid, packaging applications, functional properties

## Abstract

Petroleum based thermoplastics are widely used in a range of applications, particularly in packaging. However, their usage has resulted in soaring pollutant emissions. Thus, researchers have been driven to seek environmentally friendly alternative packaging materials which are recyclable as well as biodegradable. Due to the excellent mechanical properties of natural fibres, they have been extensively used to reinforce biopolymers to produce biodegradable composites. A detailed understanding of the properties of such composite materials is vital for assessing their applicability to various products. The present review discusses several functional properties related to packaging applications in order to explore the potential of bamboo fibre fabric-poly (lactic) acid composites for packaging applications. Physical properties, heat deflection temperature, impact resistance, recyclability and biodegradability are important functional properties of packaging materials. In this review, we will also comprehensively discuss the chronological events and applications of natural fibre biopolymer composites.

## 1. Introduction

Petroleum based polymers have been utilized as one of the main materials in a vast range of applications, gradually displacing conventional materials. The growth of plastic is mainly owing to its excellent characteristics and processing possibilities. However, the packaging industry is facing major challenges such as diminishing availability of petrochemical resources, increases in their price and the persistence of these materials in the environment beyond their functional life. Thus, this motivates packaging manufacturers to find new solutions in producing renewable and environmentally friendly products such as fibre-reinforced recyclable and biodegradable packaging materials. Green composites, defined as biodegradable biopolymers reinforced by natural fibres, have very low impact on our environment and thus they are one of the potential alternatives to replace conventional petroleum based polymers and polymer composites [1,2,3].

Due to environmental concerns and the need for improvement in functional properties, replacement of petrochemical based thermoplastics by biopolymers has been widely investigated. Poly (lactic) acid (PLA), produced from renewable resources, has received much attention as a biodegradable polymer for packaging. Factors such as increased availability of PLA and increasing petroleum costs have led to the production of PLA-based biocomposites that can compete with the petroleum-based plastics that are available in the market [4]. PLA is a good candidate for packaging applications because of its excellent mechanical properties, transparency, and biodegradability, resistance to fats and oil and its renewable resources. It resembles polystyrene in some of its properties. However, it has some shortcomings such as low heat distortion temperature, which makes its application impractical for hot food packaging, brittleness and low impact strength, low viscosity, low thermal stability, high moisture sensitivity, medium gas barrier properties, high cost (compared with polyethylene (PE), polypropylene (PP), polystyrene (PS)) and low solvent resistance (e.g., water). The above mentioned disadvantages of the pure polymer can render it unsuitable for many applications [5].

Recently, PLA has been widely used as a matrix in biocomposites with natural fibres as reinforcement. However, the trend in most of these studies is the use of reinforcement in a form of short or unidirectional natural fibres. This trend has begun to change with the use of natural fabrics to reinforce PLA composites in order to explore more efficient composites in terms of stiffness and strength. The natural fibre fabric is introduced in order to improve certain limitations in the biopolymer, such as its impact strength, brittleness and heat deflection temperature.

This review article discusses the use of natural fibre based fabric-biopolymer composites, with the emphasis on PLA and bamboo fabric as material for packaging applications. The novelty of these materials is that, unlike other common natural short fibre based composites produced for commercial packaging, they are produced from bamboo in a fabric form. This article discusses several functional properties related to packaging applications in order to explore the potential of the bamboo fibre fabric-poly (lactic) acid composites in packaging applications. Physical properties, heat deflection temperature, impact resistance, recyclability and biodegradability are important functional properties for packaging. The recent applications of natural fibre-biopolymer composites are also presented in this article.

## 2. Natural Fibres

### 2.1. Advantages and Limitations of Natural Fibres

The growing interest in natural fibres is mainly due to their low density, which is typically 1.25–1.50 g/cm^3^ compared to glass fibres with a density of 2.6 g/cm^3^, allowing natural fibres to provide higher specific strength and stiffness in plastic materials [6]. The other key driver in substituting natural fibres for glass is the lower price of natural fibres (200–1000 US$/tonnes) compared to glass (1200–1800 US$/tonnes) [7]. Natural fibres also offer several advantages as they are recyclable, biodegradable, abundant, exhibit good mechanical properties, provide better working conditions and are less abrasive to equipment compared to common synthetic fibres, which can contribute to significant cost reductions [8]. All these characteristics make their use very attractive for the manufacture of polymer matrix composites.

However, researchers who have worked in the area of natural fibres and their composites agree that these renewable sources have some drawbacks, such as poor wettability, incompatibility with some polymeric matrices and high moisture absorption [8,9]. The key differences between natural fibres and glass fibres are shown in Table 1. The most common problem of natural fibre composites is fibre-matrix adhesion. The poor adhesion between hydrophobic polymers and hydrophilic fibres of the natural fibre reinforced polymer composites can lead to lower mechanical properties. Therefore, a good bond between the matrix and fibres is essential. In a fibre reinforced composite, the matrix plays an important role in transferring the applied load to the stiff fibres through shear stresses at the interface. The full capabilities of the fibres in the composite cannot be exploited if the adhesion is poor at the interface, as well as leaving the composite susceptible to environmental attacks that may weaken it, thus reducing its life span. These properties may be improved by physical and chemical treatments [10,11].

**Figure 1:**
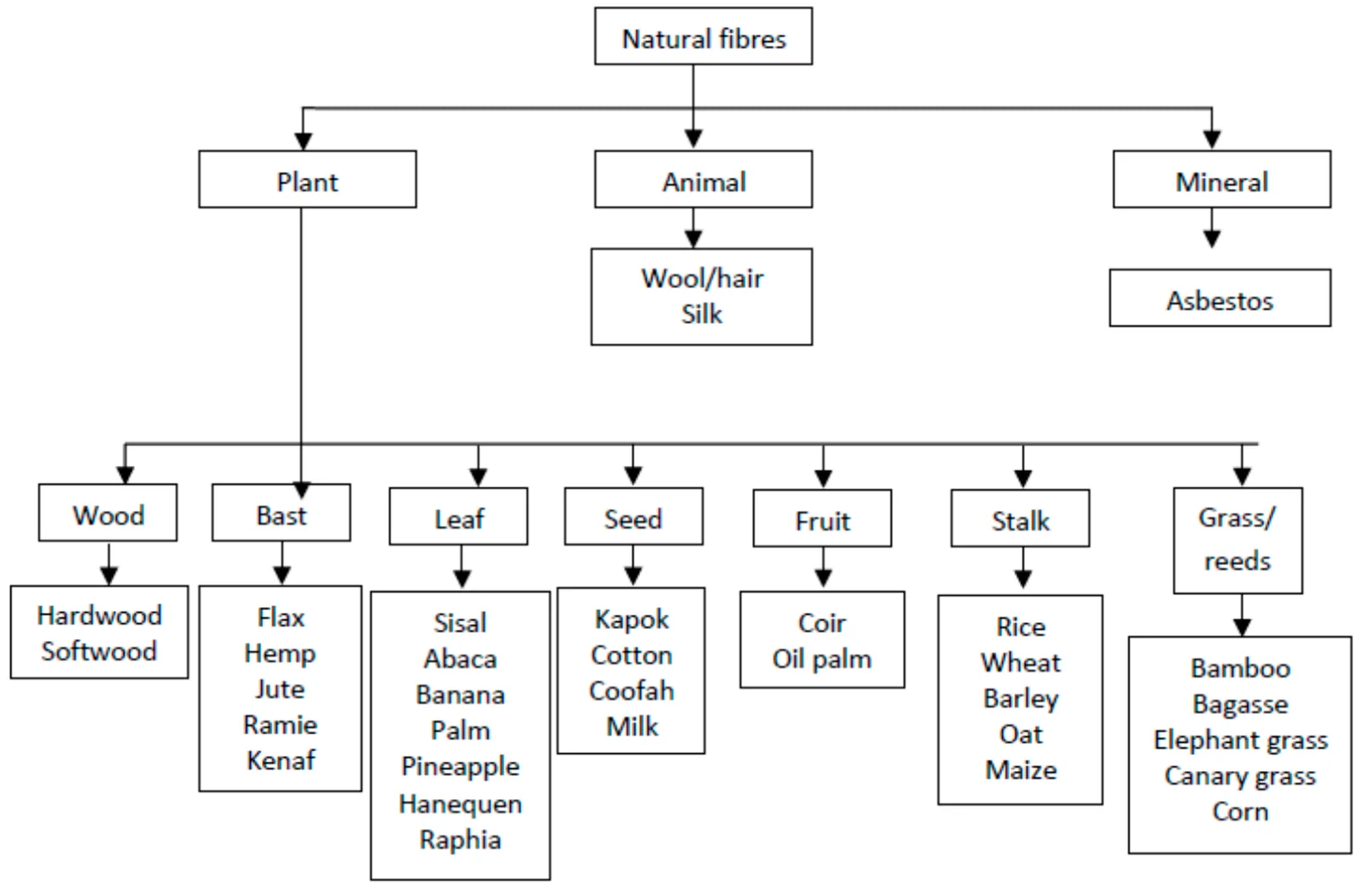
Categories of natural fibres [7,9,18].

Cellulosic fibres are hydrophilic and susceptible to moisture which can affect the mechanical and physical properties as well as lead to dimensional variations of the composites. Their moisture content can vary between 5% and 10%. The other disadvantages of natural fibres are moisture sensitivity, heterogeneous quality, and low thermal stability. The thermal degradation of natural fibres is a three-stage process. The first stage, occurring from 250 to 300 °C, is attributed to the low molecular weight components such as hemicellulose. The second decomposition process is seen in the temperature range 300–400 °C and third one is observed around 450 °C. The second degradation process is associated with degradation of cellulose whereas the degradation occurring in the last stage is due to lignin breakdown [13,14]. The degradation of natural fibres is a crucial aspect in the development of natural fibre polymer composites and can limit the use of some thermoplastics. Thermal degradation of the fibres also results in production of volatiles at processing temperatures above 200 °C. This can lead to porous polymer products with lower densities and inferior mechanical properties [15,16]. The key advantages and disadvantages of natural fibres are shown in Table 2. A wide variety of different fibres can be applied as reinforcements or fillers. Natural fibres have three main categories depending on their origin, as shown in Figure 1.

### 2.2. Why Bamboo?

**Figure 2:**
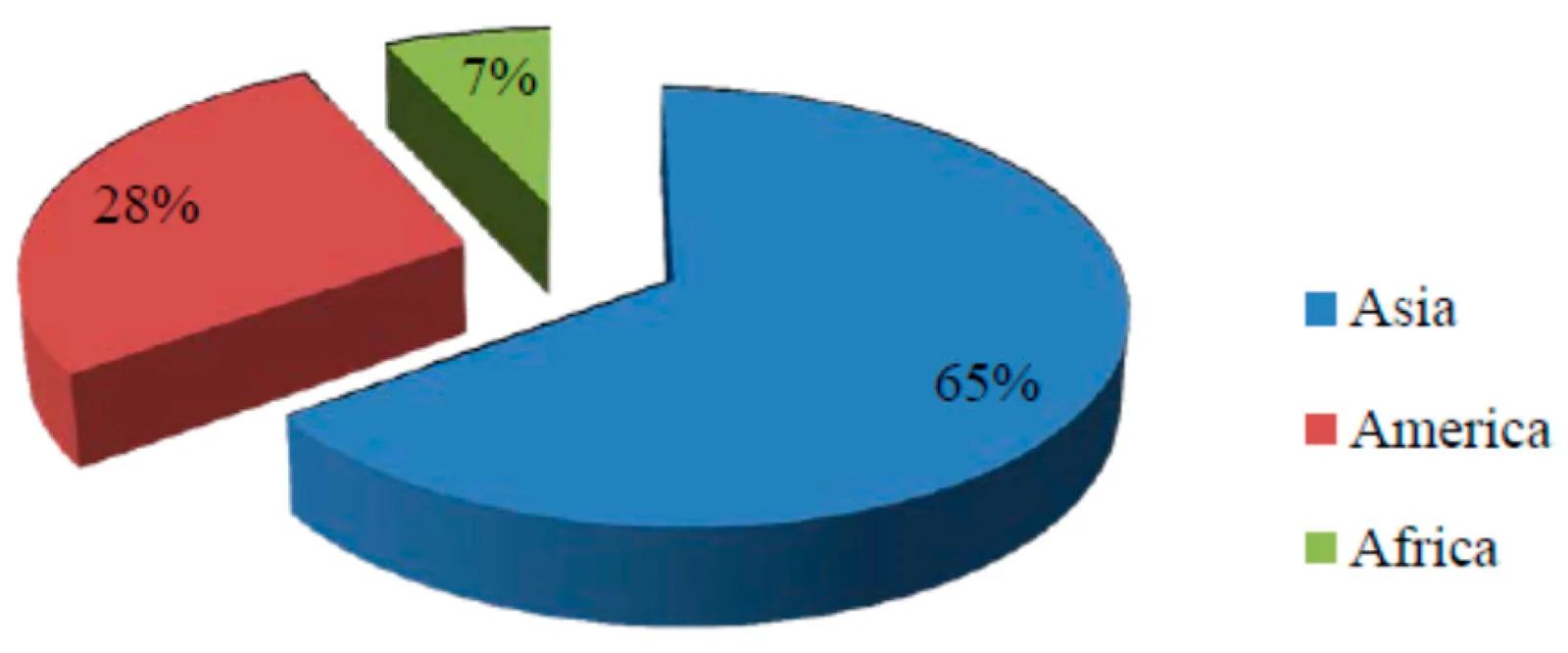
Percentage of bamboo produced by continent [21,30].

**Figure 3:**
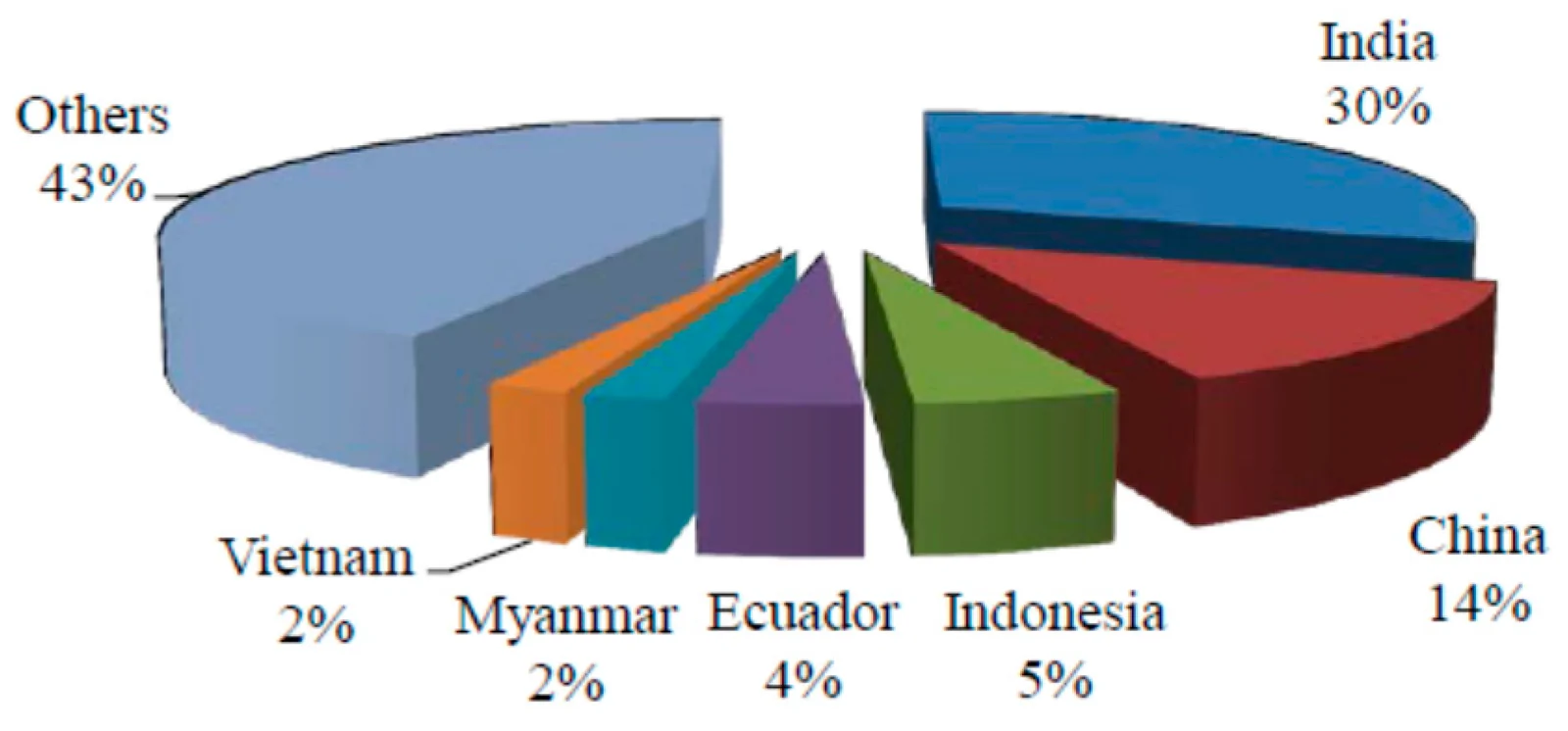
Countries with the largest bamboo resources [21].

Bamboo is one of the most under-utilised natural resources and it is available abundantly in Southeast Asian countries [19,20]. The percentage of bamboo produced by continent is shown in Figure 2, while Figure 3 indicates the countries with the largest bamboo resources. Malaysia contributes to about 1.9% of the world’s bamboo resources [21]. In particular, the bamboo stock in Malaysia is approximately 7 million tons with only 6000 tons of commonly used species [22]. Han *et al.* [19] reported that the total bamboo forest area in the world reached 22 million hectares in 2008 and worldwide availability of bamboo fibre is over 30 million tons per year.

Bamboo has attracted worldwide attention as a potential reinforcement for polymer composites because it is one of the fastest growing renewable plants, with a maturity cycle of 3–4 years. Its fibres possess excellent mechanical properties such as high tensile modulus and low elongation at break; their specific stiffness and specific strength are comparable to those of glass fibres [23]. The potential of this material for traditional composite panel manufacture has been explored. However, much of the current research focuses on composites reinforced with short bamboo fibres [13,23,24,25,26,27], and little work on bamboo fabric composites have been published [28,29].

#### 2.2.1. Chemical Composition of Bamboo Fibres

**Figure 4:**
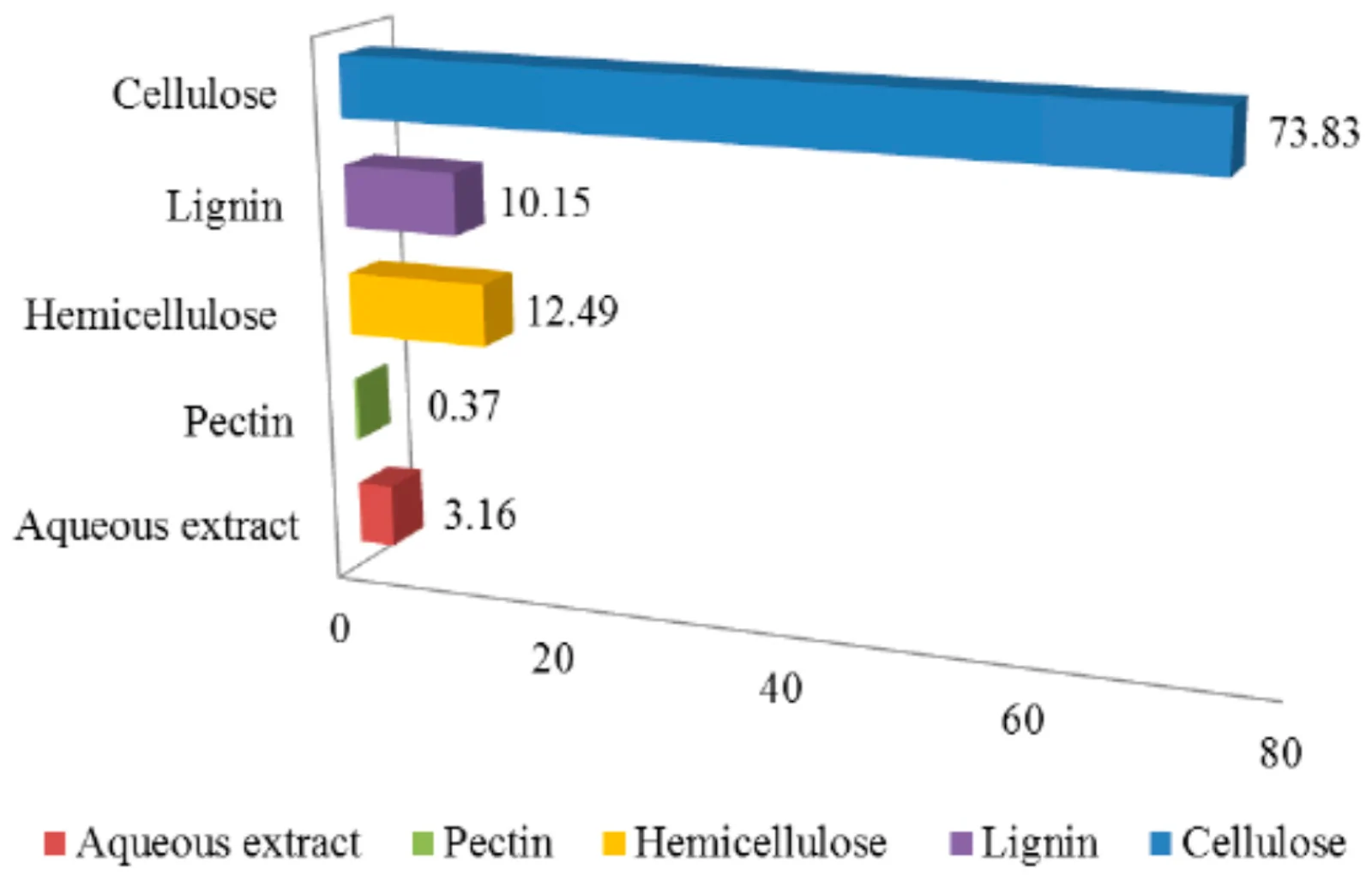
Chemical composition of bamboo fibre [30,32].

Cellulose, hemicellulose and lignin are the major compositions of bamboo which contributes more than 95% of the bamboo total mass. The other minor components of bamboo are resins, tannins, waxes and inorganic salts. Its chemical composition is similar to that of wood; however, bamboo has a higher content of minor components compared with wood [31]. The chemical composition of the bamboo fibre is shown in Figure 4.

**Figure 5:**
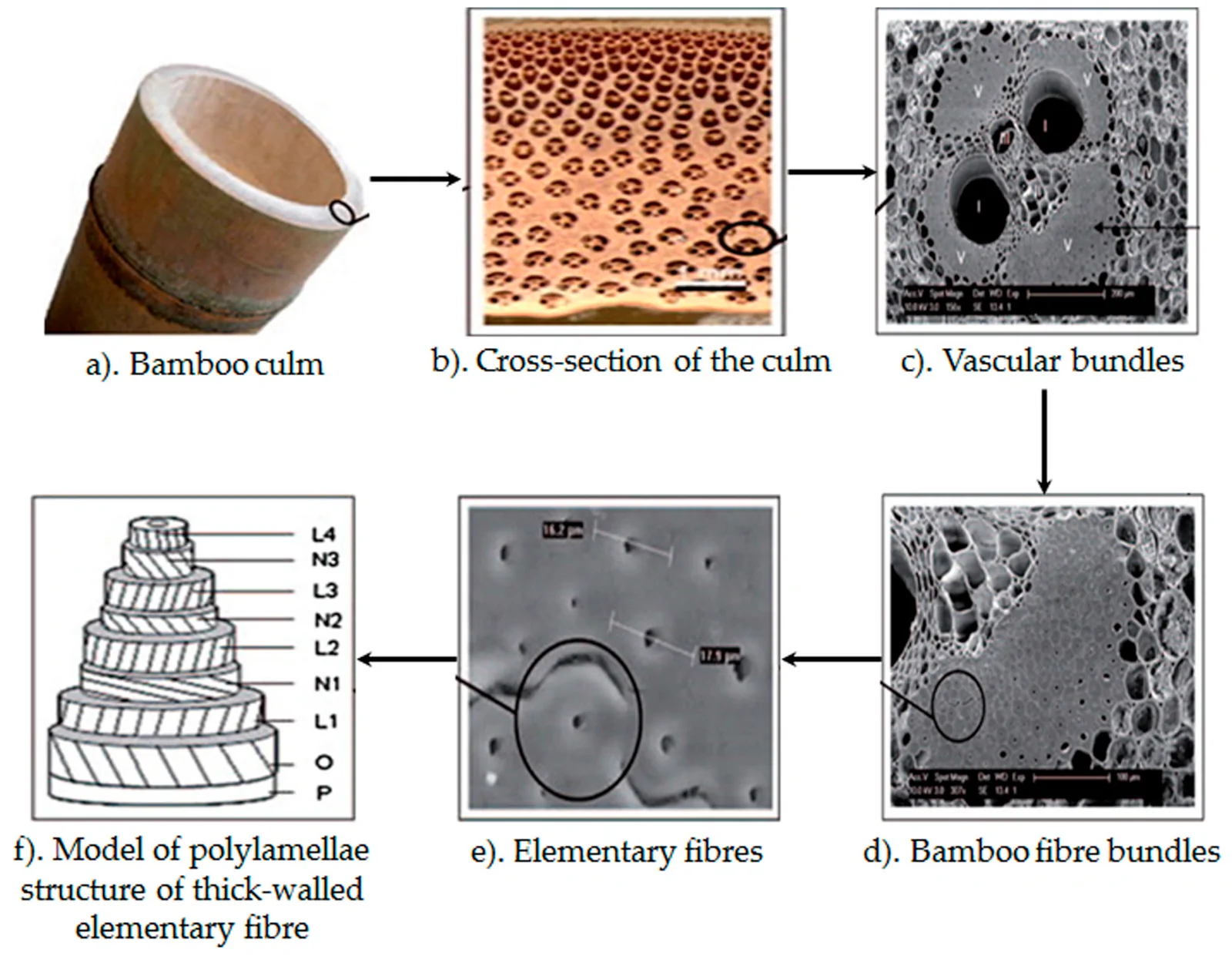
Bamboo microstructure [33].

Abdul Khalil *et al.* [30] reported that cellulose content decreases continually as bamboo ages, affecting the chemical composition of the fibres. Figure 5 illustrates the interesting microstructure and macrostructure of the bamboo which contribute to its structural reliability. Elementary fibres in the fibre bundle comprise of thick and thin layers with different fibrillar orientation. The alternate thin and thick layers have different arrangements of cellulose microfibrils. Thin layers show mainly a more transverse orientation, whereas thick layers show low microfibril angle to the fibre (Figure 5f). Lignin is present in different concentrations in different layers of the cell wall. Lignin and cellulose together provide a structural function in plants. In addition, hemicellulose and phenolic acids are responsible for covalent bonding in the cell wall structure [30,33].

The chemical compositions of bamboo, such as carbohydrate and starch content play an important role in the service life as well as the durability of bamboo against mould, fungal and borer attack. The species and climatic condition are the other factors that affect the durability of bamboo. Certain extractive could be an advantage for decay resistance in some species, while higher silica and ash content in bamboo can have an undesirable effect on the processing machinery [31].

#### 2.2.2. Mechanical and Physical Properties of Bamboo

By nature, bamboo itself is a unidirectional fibre reinforced composite comprising of long and parallel cellulose fibres (vascular bundles) surrounded by a ligneous matrix. Compared to its transverse direction, the longitudinal direction is more than ten times stronger because of its significant anisotropy in strength [33]. The fibres have a density of 1.16 g/cm^3^, while the ground tissues have a much lower density of 0.67 g/cm^3^. In bamboo, the density distribution in radial position in the cross-section area and along the length of the plant varies. The fibre density of the inner part is much lower than the outer part and the mean fibre density in the section greatly affects compressive strength of bamboo. Bamboo can be processed into various forms, such as whole bamboo, sections of bamboo, bamboo strips and bamboo fibres [34]. When compared with the other natural fibres, bamboo has high specific stiffness and strength due to its low density and high mechanical strength (Table 3) [33]. Table 3 compares a number of physical and mechanical properties of different natural fibres to those of common synthetic fibres.

## 3. Fabrics

### 3.1. Hierarchy of Textile Materials

Initially the term “textile” referred to woven fabrics; however, the term now applicable to fibres, filaments and yarns, natural or synthetic, and most products derived from them. This definition introduces three essential concepts which are fibre, yarn and fabric: (1) fibres at the *microscopic* scale; (2) yarns, repeating unit cells and plies at the *mesoscopic* scale; and (3) fabrics at the *macroscopic* scale. Each level is defined by a characteristic length, dimensionality and structural organisation. Fibres and yarns are mostly one-dimensional, while fabrics are two or three dimensional [37].

A *fibre* is defined as a raw material of textile, normally characterised by flexibility, fineness and ratio of length to thickness. The diameter of fibres used in textile reinforcements for composites (glass, carbon, aramid, polypropylene, flax, *etc.*) varies from 5 to 50 µm. Fibres of finite length are called *short*, *discontinuous*, *staple* or *chopped* with lengths from a few millimetres to a few centimetres while continuous fibres are called *filaments*, which are effectively infinite in length [37,38]. *Yarn* is a linear assemblage of fibres and fibre plies; which is then used to produce textile. A *textile* fabric is defined as a manufactured assembly of fibres and/or yarns. The properties of a fabric are the properties of fibres transformed by the textile structure.

### 3.2. Textiles Terminology

A yarn can be made of fibres and/or filaments, with or without twist. It has substantial length with relatively small cross-section. The simplest form of yarn is *monofilament* which consists of a single fibre. Untwisted, thick yarns called as *tows* are made by including an adhesive. This adhesive or *sizing* holds the fibres together to facilitate tows manufacturing. This adhesive can also be used as the matrix material in composites. Flat tows are called *rovings.* Spinning is a process to produce twisted yarn from staple fibres, whereas twisting is a process to produce twisted yarn from continuous filament yarn. In the spinning process, twist is the primary binding mechanism that gives the yarn a twisted structure. Twist will help to bind the fibres by generating inter-fibre friction and thus imparts processability to the yarn. *Blends* are produced from fibres of different types which are easily mixed when yarn is spun. Finally, a ply yarn can be produced by twisting several filament yarns together [37].

### 3.3. Fabric Structures

There are three main categories of fabric structure; woven, knitted and braided fabric, however, only woven fabric will be discussed here. Woven fabric has a structure in which the warp yarns and the weft yarns are interlaced. The warp direction is parallel to the length of the roll, while the fill or weft direction is perpendicular to the length of the roll [39]. The tensile strength of a woven fabric is equivalent to the sum of the strengths of the yarns oriented along the tensile direction. However, several issues related to woven fabric have been identified and studied by researchers worldwide [40]:

1. The presence of twist in the yarns will disturb the stress transfer between fibres within the yarn; subsequently this will affect the fracture mechanism and strength of the yarn.
2. The ‘crimp’ normally occurs from the utilization of twisted yarns to produce woven textile reinforcements. This phenomenon will result in misalignments and stress concentrations that will lead to an unfavourable effect on composite properties.
3. The yarn permeability and impregnation are reduced because the twist tightens the yarn structure.

**Figure 6:**
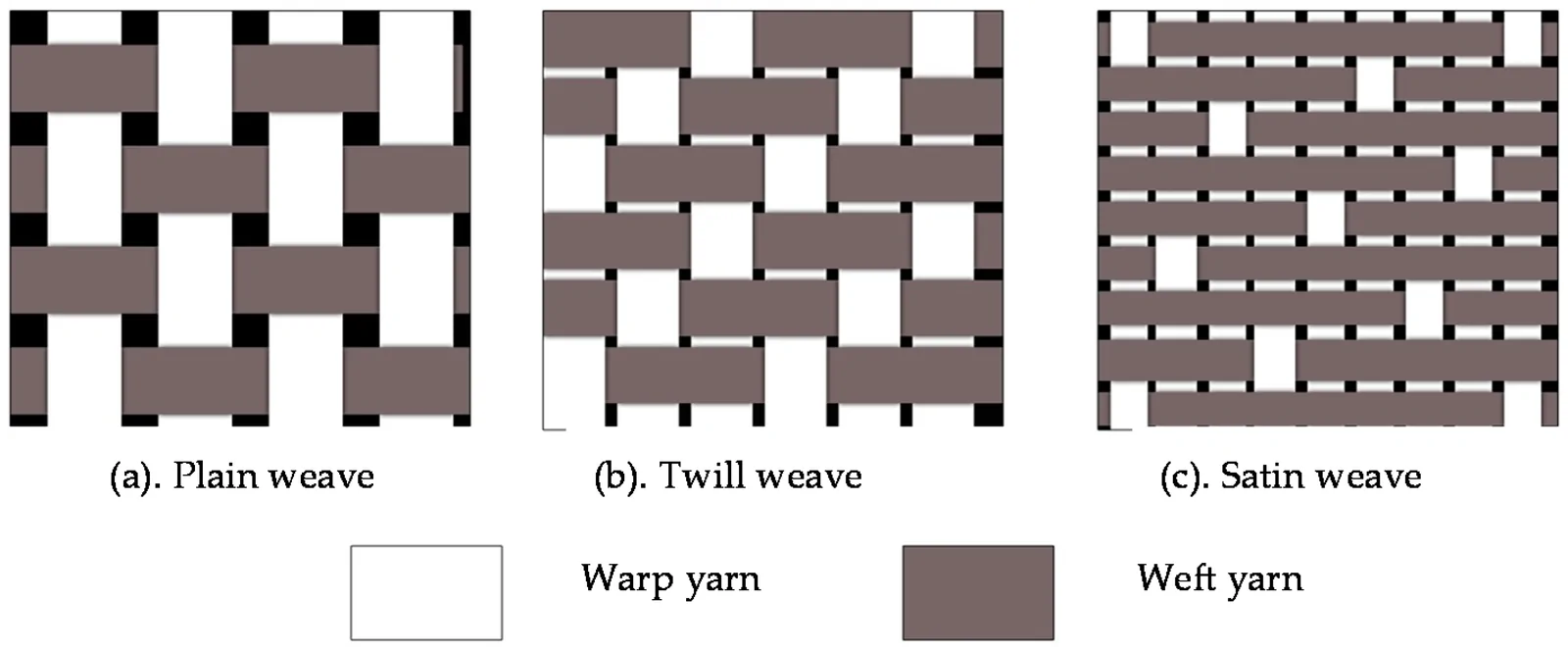
Woven fabric terminology.

Woven fabric is identified by the linear densities of warp and weft yarns, the weave pattern, the number of warp yarns per unit width (warp count), a number of weft yarns per unit length (weft count), warp and weft yarn crimp, and surface density. The simplest woven fabric structure is the *plain weave* where the warp and weft yarns are interlaced in a regular sequence of one under and one over. Plain weaves have more interlaces per unit area than any other type of weave, and therefore the tightest basic fabric design, and are the most resistant to in-plane shear movement [41]. The difference between each types of woven fabric can be seen in Figure 6.

*Twill weave* is defined as a fundamental weave with yarns are interlaced to produce a pattern of diagonal lines on the surface. *Satin weave* is a fundamental weave characterised by sparse positioning of interlaced yarns, which are arranged with a view to producing a smooth fabric surface devoid of twill lines (diagonal configurations of crossovers).

### 3.4. Bamboo Fibre Fabrics

There are two key advantages that can be obtained by manufacturing textile from bamboo. First, the benefits resulting from the utilization of the bamboo plant itself: its abundance and low cost as a natural resource, its renewability, its efficient space consumption, its high takes up of greenhouse gases and oxygen release, the fact that it does not need replanting and requires no pesticides or fertilisers, its low water use, and its organic status. Bamboo also yields 50 times as much fibre per acre as cotton. Second, benefits resulting from fabric properties given from the plant such as its natural antibacterial and biodegradable properties, as well as UV protective characteristics [32,42]. Bamboo fibres also possess many excellent properties when used as textile materials, such as high tenacity, high strength and stiffness due to continuous fibres oriented on at least two axes [43]. Moreover, the yarn is only need to withstand the stresses experienced during the manufacturing of the fibre reinforced composites. This is because the interfacial bonding between the fibres and matrix will replace the fibre-to-fibre friction created by the twist [44].

However, the essential factors in the textile industry such as the energy, water, and chemical requirements that can be involved in its manufacture are the main restrictions of bamboo textiles [45]. Table 4 reveals the chemical components of flax, jute and bamboo fibres after degumming through a chemical treatment process. The cellulose content in the bamboo fibre reaches more than 70%, which meets the preliminary requirement for textile applications [32].

#### Fabric Processing Method

The cellulosic fibre extraction of bamboo by degumming process is challenging due to the main components in bamboo invades each other and forms an interpenetrating network structure. Therefore, a more intensive degumming effect for fibre extraction is required because of the extremely tight structure of bamboo culm. Ramie has a long fibre (length varying from 60–250 mm), which is suitable for downstream steps in textile processing. Meanwhile, bamboo (monofilament) has shorter fibres, which is between 1.5 and 4.5 mm in length. Thus, different degumming methods should be used to process bamboo fibres for textile utilization. Similar technique is also required for other plant like hemp, kenaf and flax to obtain processable fibre bundles for textile application [47].

Several different manufacturing processes can be used to convert bamboo from the plant to the woven fabric, with different effects on the environment. Currently, there are two main manufacturing methods for bamboo textiles: Mechanical processing (stripping, boiling, and enzyme use) is similar to flax processing and produces linen-like fabrics; Chemical processing (multi-phase bleaching) is quite similar to that of viscose rayon fibre and produces fabrics similar to rayon from bamboo.

(a) Mechanical processing

Mechanical methods of producing bamboo fibre fabric are less harmful but more expensive. In this process, the bamboo is crushed, followed by the utilization of natural enzymes to breakdown the bamboo walls into a soft form. In the soft form, these fibres can be mechanically combed out and spun into yarn. Since this process is more labour intensive and costly, it is hardly used for manufacturing clothing with bamboo fibre. This process will produce a relatively stiff and rough bamboo fabric like flax (linen) or hemp [30].

(b) Chemical processing

Most bamboo fabric in the market has a smooth-textured that feels similar to rayon; in fact, it is a form of rayon. Through chemical process, the bamboo can be converted into a regenerated cellulose fibre known as rayon, which falls into a category between natural and synthetic. The viscous process is an established and widely used process for bamboo. In the chemical process, a strong solvent is used to dissolve the cellulose material into a thick solution. Then, this solution is forced through a spinneret into a quenching solution in order to solidify the strands into fibre. This process is also known as hydrolysis alkalization or solution spinning. The solvent used for this process is carbon disulfide, which is harmful for human reproductive and pollutes the environment through air emissions and wastewater [30]. The recovery of this solvent is normally around 50%; the other half goes into the environment. Sodium hydroxide and sulfuric acid are the other hazardous chemicals used in the viscose process [48]. There is an environmentally friendly chemical process also known as closed loop system called lyocell. The only weakness for this method is the high cost in setting up the factories [49].

(c) Biological degumming

Recently, the utilization of biotechnology in textile industry is becoming more important especially in bamboo textile manufacturing. Single biological treatment, either microbial or enzymatic treatment has been established as an effective treatment to remove specific substances from lignocellulose materials. For bamboo degumming, in order to achieve efficient degradation or removal of unwanted component, a combination of a pre- and post-treatment is essential. This is because bamboo consists of high lignin content which hinders the attack of microbes or enzymes. However, the biological treatments are very limited when applied directly on raw materials due to an extremely tight structure of bamboo itself. Therefore, biological methods are normally combined with mechanical or chemical treatments for bamboo fibre extraction. There are two biological degumming methods identified; microbial and enzymatic degumming. The combination of microbial and enzymatic degumming has also been investigated by many researchers [47].

##### Microbial Degumming

For microbial degumming, it comprises of a microbial consortium or mixed microbial cultures which are directly inoculated in the plant material. The gummy substances will be utilized by the microbes as a source of nutrition and further grow on a large scale in the plant resources. Then, the enzymes can be accumulated from the microbes during growth and activate the degradation of the gummy materials into small water soluble molecules [47].

##### Enzymatic Degumming

In the case of enzymatic degumming, purified and concentrated enzymes or commercial enzymes from microbial fermentation are applied directly to the plant material in an appropriate buffer. The fibres are separated when the enzymes initiate the degradation of the colloid complexes. Thus, selection of the right enzymes to achieve the highest degumming yield is vital for an effective bio-degumming [47].

## 4. Biopolymers

**Figure 7:**
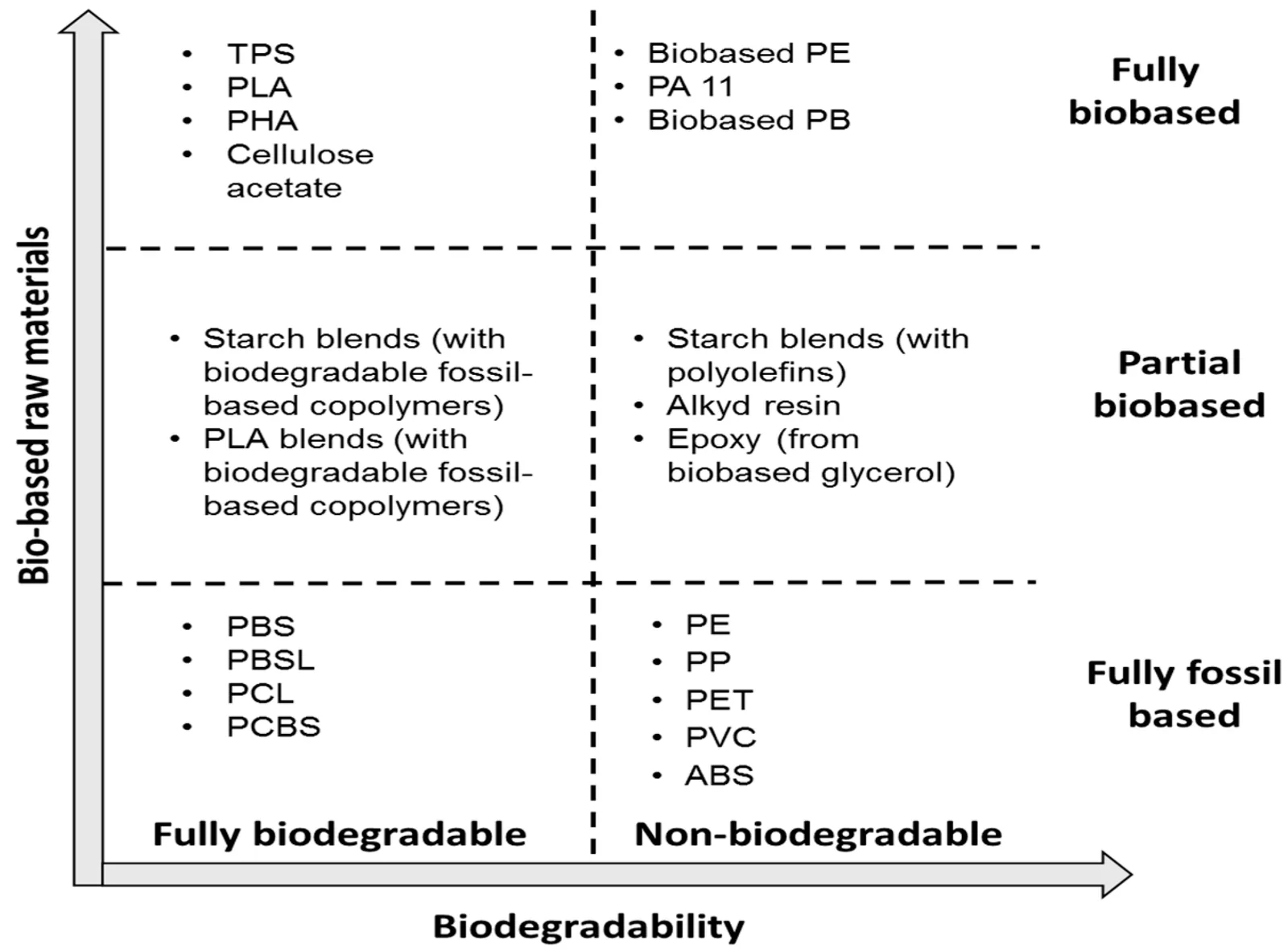
Schematic representation of the diversity of degradable materials [53].

Biopolymers are polymers that are generated from renewable natural sources, are often biodegradable and nontoxic. Biodegradable materials are defined as those materials which can be degraded by the enzymatic action of living organisms that produce CO_2_, H_2_O, and biomass under aerobic conditions or hydrocarbons, methane and biomass under anaerobic conditions [50,51]. They can be manufactured from biological systems such as microorganisms, plants, and animals, or chemically synthesized from biological materials such as starch, natural fats, sugars or oils. Two approaches are used in converting these raw materials into biopolymers: (1) extraction of the native polymer from plant or animal tissue; and (2) chemical or biotechnological monomer polymerization [52]. Figure 7 schematically illustrates the variety of biodegradable plastics.

### Why Poly (Lactic) Acid (PLA)?

**Figure 8:**
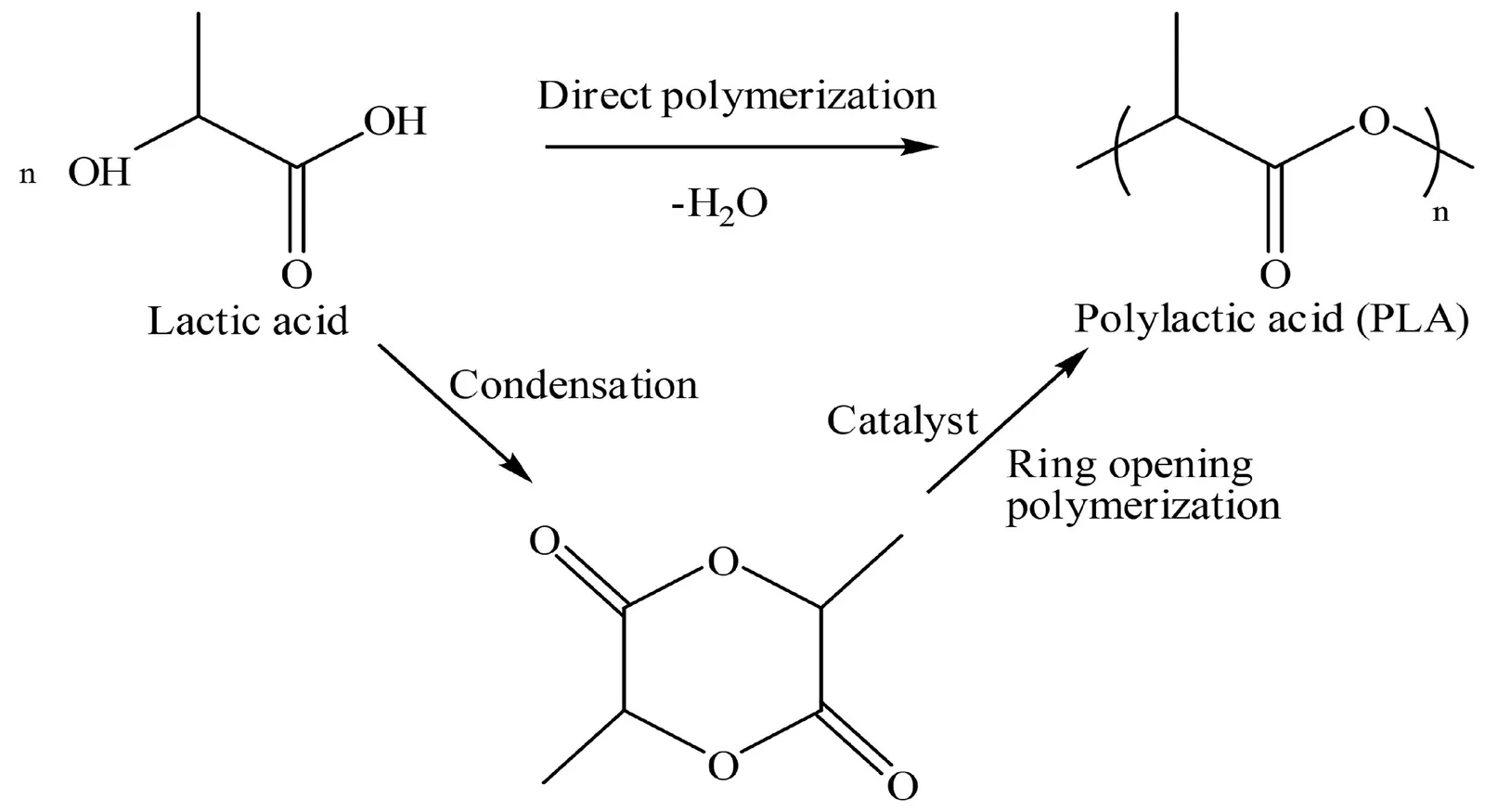
Synthesis of poly (lactic) acid (PLA) [54].

Poly (lactic) acid (PLA) which is produced from renewable resources, has received much attention in the research of biodegradable polymers. The feedstock from crops such as corn and sugarcane is fermented to obtain lactide and lactic acid monomers. Two-step ring-opening polymerization of lactide is the most common method to acquire the high molecular weight of PLA (greater than 100,000 Da). In 2002, *Cargill Dow* used about 300 million USD to begin mass-producing its new PLA based plastic under the trade name NatureWorks™ [52]. Figure 8 presents the synthesis process of PLA.

There are several advantages to PLA, such as it is obtained from a renewable agricultural source (corn), its production utilizes carbon dioxide and offers significant energy savings, it is recyclable and compostable (under the right conditions), as well as its physical and mechanical properties can be manipulated through the polymer architecture [55].

However, a drawback of processing PLA in the molten state is its tendency to undergo thermal degradation, which influences its process temperature and the residence time in the extruder and hot runner. This affects the recyclability of PLA [56]. Several reports have shown that PLA is thermally unstable above its melting temperature. The presence of moisture has a particularly significant effect at higher temperatures as it induces hydrolysis. Thus, care has to be taken to ensure that PLA does not undergo degradation during processing. Drying samples before use or processing under nitrogen atmosphere is recommended [57].

Currently, research related to PLA as a matrix in natural fibre biocomposites is relatively limited because only recently PLA has been available in bulk quantities. The cost of PLA is comparable with polyethylene terephthalate (PET) but quite expensive when compared with other commodity thermoplastics such as polyethylene and polypropylene. Due to its high cost, the primary attention of PLA as a packaging material has been in high value films, rigid thermoforms, food and beverage containers and coated papers. However, as evolving production technologies may lower the PLA production costs, PLA could be used in broader range of packaging products.

“Green composites” that are produced from PLA reinforced natural fibre composites can provide promising mechanical properties in comparison to non-renewable petroleum-based products [54]. Furthermore, fibre addition can also enhance properties of PLA such as heat deflection temperature, impact strength and barrier properties.

## 5. Natural Fibre Fabric Composites

The packaging industry is looking for materials which have specific properties, but can also be easily thrown out after usage [58]. Each component in “green composites” originates from renewable resources that conserve energy and it is compostable [54,59] which makes it a potential eco-friendly material for packaging applications. Research related to natural fibre fabric-biopolymer composites have been carried out by several researchers. Table 5 summarises the mechanical properties of natural fibre fabric-PLA composites research papers available to date.

## 6. Packaging

Packaging can be defined as the process of preparing items for preservation, storage, transportation and display. Packaging also must meet requirements such as [9]:

- the ability to keep the item safe from physical and chemical damage
- the ability to provide ease during handling and transportation
- the ability to encourage consumers to purchase the product

There are three categories of packaging: primary, secondary and tertiary packaging. Primary packaging can be defined as material that come into direct contact with but can be separated from the product. Secondary packaging is used for physical protection of the product, while tertiary packaging fulfils storage and handling requirements of the product as well as to protect the product from damage and weather conditions during transportation [9].

Current development necessity for environmentally friendly packaging designs and materials is being driven by an increasing number of global packaging standards and regulations. Based on the Sustainable Packaging Coalition, packaging should be “sourced responsibly, designed to be effective and safe throughout its life cycle, made entirely using renewable energy and once used, is recycled efficiently to provide a valuable resource for subsequent generations of packaging” [66]. Plastic packaging is commonly used once and then thrown away, generating a continuous waste stream. Formerly, the use of natural fibres as fillers in non-biodegradable polymers or the use of biodegradable polymers as packaging materials have been the main approaches to reduce this waste [9].

**Figure 9:**
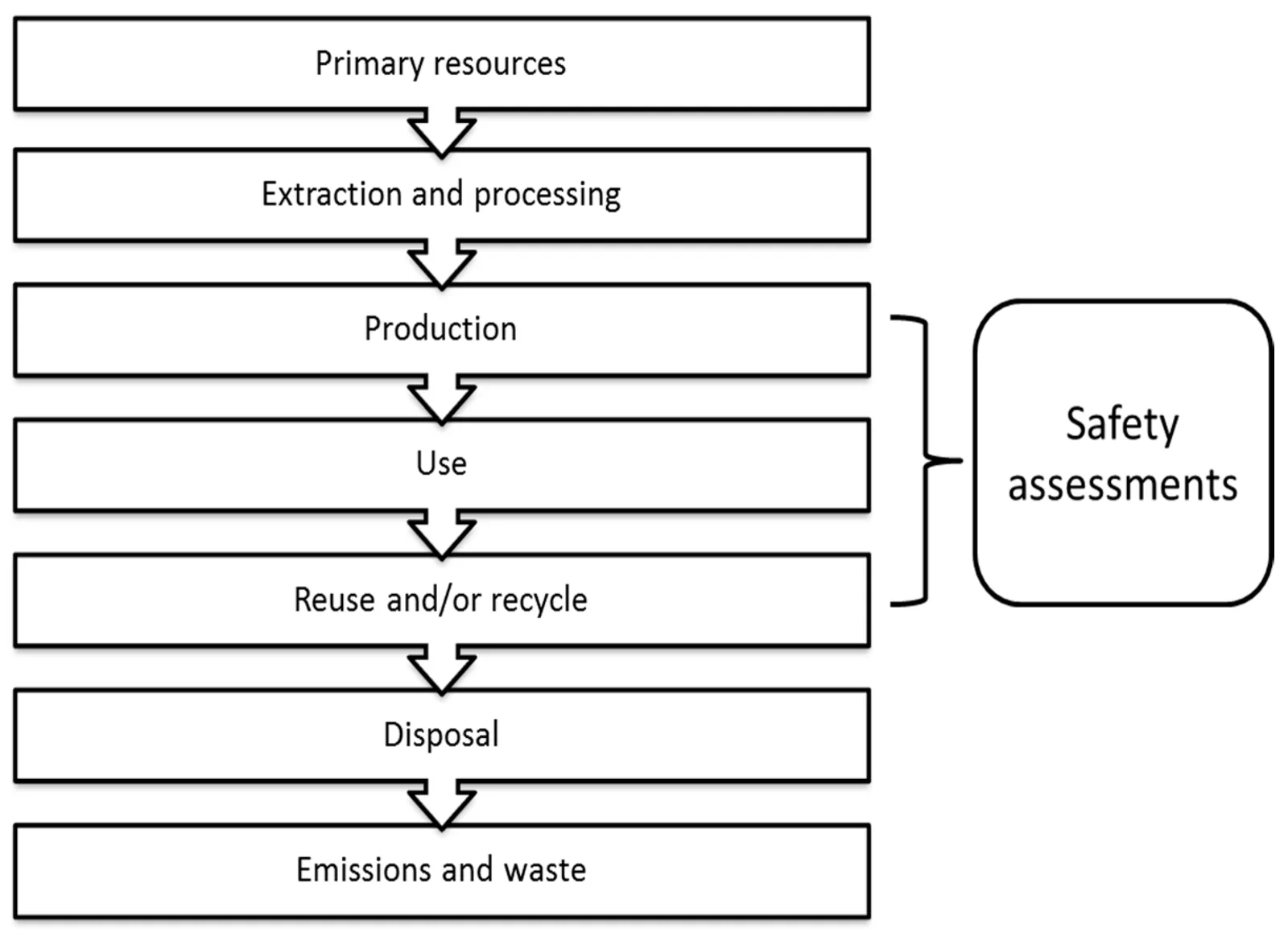
Life cycle of the packaging [67].

The life cycle of the packaging starts with the selection of the raw materials, depending on the types of packaging product, followed by a suitable production process. Then, the packaging products will be used by the consumers, who decide whether to reuse or recycle them. For biopolymer packaging materials, the consumers will have another option; to collect the materials to be degraded via a biodegradation process under controlled conditions. The complete life cycle of the packaging is shown in Figure 9.

### 6.1. Properties Related to Packaging Applications

Several properties related to packaging application have been identified. These properties are important to provide an understanding of the materials in conversion into packaging products. The properties can be divided into three categories; mechanical, thermal and functional properties.

#### 6.1.1. Mechanical Properties

Mechanical properties are important in order to predict how the materials behave under loads when they are subjected to various manufacturing operations. These properties also demonstrate how finished products will perform during their entire service life. Several mechanical properties of natural fibre fabric reinforced PLA composites are shown in Table 5.

#### 6.1.2. Thermal Properties

An understanding of the thermal properties of these composites is necessary in determining appropriate packaging applications. Composite thermal properties, such as glass transition temperature and melting temperature, affect the mechanical behaviour of composites and therefore, are influential in determining suitability of composites for certain packaging applications. Glass transition temperature is very critical when the packed material is to be stored in a frozen environment. The manufacturer must ensure that the glass transition temperature of packaging material is lower than the freezer temperature. Otherwise, the packaging material will become brittle, and may crack [68]. Nurul Fazita *et al.* [69] reported the thermal properties, such as melting temperature, glass transition temperature and heat deflection temperature of bamboo fabric-PLA composites as the important properties for packaging applications.

#### 6.1.3. Functional Properties

Functional property evaluation of the bamboo fabric-PLA composites can be achieved by determining whether the manufactured composites show any improvement in their properties when compared with the unreinforced polymers. Physical properties, heat deflection temperature, impact resistance, recyclability and degradability are the functional properties that are relatively important for packaging products.

##### Physical Properties

Appropriate selection of materials and the specific manufacturing processes are crucial for the proper design and manufacturing of a product. These factors will further affect the service life of the finished products. Thus, physical properties such as density, moisture content, thickness and water absorption are essential for packaging materials.

Thickness uniformity is an important criterion in packaging product manufacturing. On the other hand, knowledge of the moisture properties of packaging materials is essential for providing an understanding into the mechanics of failure and the roles of materials in the failures. This is important for material selection, failure analysis, as well as material development. One of the most important issues for natural fibre thermoplastic packaging is the poor resistance of the fibres to water absorption. This can have undesirable effects on the mechanical properties and dimensional stability of the composites. The water absorption test is essential to determine the quantity of water absorbed by a material as well as the effects that the water absorbed may have on the appearance of the composites. Nonhomogeneous materials such as laminated form may exhibit widely different rate of water absorption through edges and surfaces.

##### Impact Resistance

Many research works have reported the impact strength of natural fibre-PLA composites using Charpy and Izod impact testing [5,70,71,72,73]. These traditional impact test methods give inadequate information to understand the impact fracture behaviour of materials because they only provide the total energy consumed during the entire impact fracture process. Impact damage of polymeric composite materials is a complex process [74].

The instrumented impact test is a sophisticated method for measuring impact properties. It provides more information about relationships relevant to toughness such as impact force-time, velocity-time, energy-time and force-displacement. The drop weight impact test is now widely used to study the impact behaviour of composite materials. In this test, a sample is positioned on rigid supports and then the sample is impacted with a rod of certain weight from a desired height. The drop weight impact test is conducted to study the susceptibility of the composites to damage from concentrated out-of-plane impact forces [75]. Drop weight impact has some important advantages over other methods such as its applicability to moulded samples. Failure can be defined by deformation, crack initiation and propagation, or complete fracture, depending on the requirements; samples do not have to break to be considered as failures. These factors make drop weight testing a reliable simulation of functional impact exposures, and therefore closer to real-life conditions.

Understanding the behaviour of woven fabric-reinforced composites in an impact event by studying the formation of damage and analysing the absorbing effects under low-velocity impact will lead to improvement in their damage-resistance characteristics and enhance their use in packaging. A few studies have been conducted using drop weight impact tests on natural fibres-PP composites [76,77,78] and limited work has been done with the natural fibre-PLA composites [69]. Several researchers have considered drop weight impact test for PLA related to their use in biomedical, structural and packaging applications [79,80,81].

##### Heat Deflection Temperature (HDT)

The deflection temperature can be defined as a measure of a polymer's ability to stand a given load at elevated temperatures and is a useful measure of relative service temperature for a polymer. HDT is considered as one of the important properties for packaging applications. It is closely related to the concern over transportation, particularly during summer months and in warmer climates where the temperature is relatively high in unconditioned environments. Low HDT is one of the drawbacks of PLA that limits its application [5,82]. The other problem associated with HDT is the resistance of the PLA packaging or containers to be “hot-filled” [83,84]. A few researchers have found that by adding fibres, the HDT of the PLA composites can be improved [82,85]; however, limited work has been done to investigate the HDT of natural fibre fabric composites [69].

##### Recyclability

**Figure 10:**
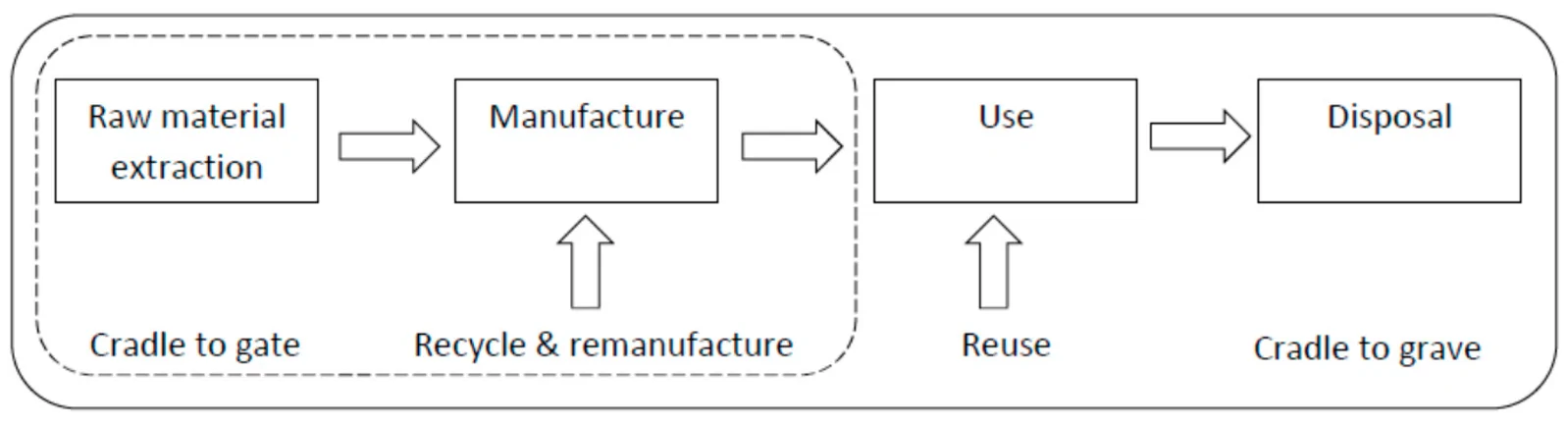
Life cycle stages.

The life cycle stages of thermoplastic composite materials are shown in Figure 10. Reuse by virtue of a recycling step is feasible for thermoplastic composites within certain restrictions. Generally, this recycling involves crushing the parts before a mixing phase to blend the recycled materials with virgin material. This mixture is then processed under high pressure and temperature by means of conventional injection or extrusion technologies [86].

Recycling of composites can minimise raw material consumption leading to a reduction in the the global impact on the environment. Mechanical and thermal degradation of both the matrix and the reinforcement will occur during the recycling. Pillin *et al.* [87] studied the thermo-mechanical effects of recycling on the mechanical properties of the PLA, noting constant Young’s modulus but with a decrease in stress and strain at break.

Degradation of PLLA can be initiated through temperature changes in the presence of air and random chain scissions will occur [59]. The structure of semi-crystalline polymers such as PLLA will also change during multiple injections. Crystallisation occurs during cooling and may be enhanced if a reduction in molecular weight results in greater mobility of molecular chains. In comparison, the crystallinity of polypropylene in hemp/PP and sisal/PP composites does not change significantly when these materials are subjected to several injections [88]. The mechanical properties of sisal/PP and hemp/PP have also been investigated as a function of recycling cycles. Natural fibres have limited thermal stability. Gassan *et al.* [89] demonstrated that the onset of thermal degradation of flax and jute fibres occurs at around 170 °C. The recyclability of bamboo fabric-PLA composites has been done by Nurul Fazita *et al.* [90]. They found that mechanical (tensile and flexural) strength and thermal stability of recycled bamboo fabric-PLA composite were higher than those of virgin PLA.

##### Biodegradation

In general, polymer degradation takes place through the scission of the main or side chains of polymers. Different degradation mechanisms, whether chemical or biological, can be involved in the degradation of biodegradable polyesters. A combination of these mechanisms can happen at some stage of degradation. There are several important factors that affect the biodegradability of polymers. These are: (1) factors associated with the first-order structure (chemical structure, molecular weight and molecular weight distribution); (2) factors associated with the higher order structure (glass transition temperature (*T*_g_), melting temperature (*T*_m_), crystallinity, crystal structure and modulus of elasticity); and (3) factors related to surface conditions (surface area, hydrophilic, or hydrophobic properties) [91].

Previous research has shown that the crystalline part of the PLA is more resistant to degradation than the amorphous part, and that the rate of degradation decreases with an increase in crystallinity. The degradation behaviour of polymers also depends on their molecular weight. High molecular weight PLA are degraded at a slower rate than those with low molecular weights. The melting temperature (*T*_m_) of PLA has a great effect on enzymatic degradability. In general, the higher the melting point the lower the degradability tends to be [91].

Ochi [92] used enzymatic composting material for the investigation of PLA/kenaf composite, and found the weight of composite had decreased 38% after 4 weeks of composting. Mathew *et al.* [93] achieved a higher degradation rate without enzymatic material by using the higher temperature of 58 °C. Yussuf *et al.* [94] obtained a lower rate of degradation for PLA/kenaf composite, attaining weight loss of only 1.2%, very slow compared to the enzymatic techniques. The very low average temperature of 30 °C used was likely the reason for the slow rate of degradation observed in this research. Rudeekit *et al.* [95] investigated the biodegradability of PLA under different environments, e.g., landfill, waste water treatment, composting plant and controlled composting conditions. PLA was degraded rapidly under composting plant conditions. This may be due to the high temperature and humidity (50–60 °C and 60%).

The degradation process of PLA under aerobic conditions in compost is started by a sequential mechanism beginning with a simple chemical hydrolysis to decrease the molecular weight of the PLA. And then, assimilation will occur when the microorganisms utilise the lactic acid oligomers as an energy source. Finally, the end product of the process is compost and release of CO_2_ back into the atmosphere. Aerobic degradation is highly dependent on temperature. At the temperature of 55–60 °C with little or no degradation at mesophilic temperatures, complete biodegradation will occur within 3–4 months. On the other hand, Kolstad *et al.* [96] reported that soil burial for a year (0–22 °C) have no effect at all on physical properties of PLA test bars. Kolstad *et al.* [96] also discussed some of the many factors which are involved, such as water content, temperature, crystallinity, pH, and other factors.

Shi and Palfery [97] reported that an anaerobic biodegradation is accelerated when the degrading temperature is greater than the PLA glass transition temperature (*T*_g_). This may be due to the fact that above *T*_g_, the amorphous part of the PLA becomes more susceptible to microorganisms. However, the anaerobic biodegradation is apparently slowed when the degrading temperature is below PLA *T*_g_. The biodegradability of bamboo fabric-PLA composites has been done by Nurul Fazita *et al.* [90]. They revealed that in controlled composting conditions, the biodegradation half-life of bamboo fabric-PLA composites (46 days) was longer than that of the virgin PLA (25 days). This shows that the reinforcement of bamboo fabric in PLA matrix minimize the rapid microbial decay of bamboo fabric-PLA composite. They also reported that the biodegradability of PLA matrix potentially depended on the presence of bamboo fabric and composting conditions.

##### Energy Absorption Capability of Textile Composites

Recently, the energy-absorbing capacity of textile composites attracted the attention of researchers. The assemblies of fibres with certain orientations and low packing density of textile structures are similar to cellular materials which possess excellent energy absorption capacity. The structural parameters of fibre assemblies, such as energy absorption can be designed for particular composite applications. This kind of materials can be used in various areas where the specific energy-absorbing capacity is the main technical concern, such as in protective packaging and crushing elements in cars and bicycle helmets [98,99].

Safety, quality and material costs require an effective testing of packaging materials to the point of collapse or deformation. Thus, the drop weight test is the most suitable test in studying the formation of damage and analysing the absorbing effects under low-velocity impact. The purpose of this test is to simulate these real-life conditions in order to prevent the product from breaking, or to make its failure predictable. This information can help to improve their damage-resistance characteristics and enhance their use in packaging. Impact testing, conducted on materials in both raw and finished states, allows the manufacturer to determine the best combination of material components. During impact tests, the materials and products are pushed to absorb loads quickly by exposing them to the dynamic events. Valuable information as to how that material will perform in real-life situations will be provided by this type of testing.

Many studies on the energy-absorbing capacity have been performed using quasi-static and impact tests. In investigating the quasi-static behaviour of the dome materials, researchers have concentrated on how the domes are deformed and distorted after the load is applied at various orientations. Meanwhile, the studies of dynamic behaviour have revealed that dynamic deformation mechanisms and energy-absorbing capacity are significantly different due to strain rate and non-local effects. The findings of the extensive research work which has been carried out in quasi-static and impact testing has demonstrated that there are multiple variables which control the energy absorption capability of the composites materials, such as fibre and matrix properties, fibre architecture (stacking sequences), and the testing speed [100,101,102].

## 7. Chronological Event of Natural Fibre Fabric-Biopolymer Composites Study and Applications

Recent advances in the field of green composites, natural fibre fabric and biocomposites indicate the importance and potential of biocomposites for numerous applications. Regardless of its noteworthy properties, the use of petroleum based composites has become unattractive due to a few reasons, including disposal and recycling problems, as well as environmental and societal concerns. Therefore, natural fibre fabric-biopolymer composites have been extensively studied recently as they provide a positive environmental impact and remarkable characteristics. The chronological order of events that took place in the exploration of natural fibre fabric-biopolymer composites study and its related applications in various fields are shown in Table 6.

## 8. Current Packaging Applications of Natural Fibre Reinforced Composites

A few companies have already started to use natural fibre composites instead of typical plastic packaging materials. This points out the fact that natural fibre fabric-biopolymer composites particularly bamboo fibre fabric-PLA composites which are entirely based on renewable resources, could find interesting packaging applications at the industrial level as environmentally friendly materials that can substitute some commodity plastics. Since natural fibre fabric-biopolymer composites have better impact properties as compared to short fibres-biopolymer composites [23], they can be used in rigid packaging or in protective packaging. These products will have satisfied performance during service and will return back to the nature after use under certain composting conditions.

### 8.1. Cosmetics Packaging

Rexam Beauty Packaging has introduced containers for a perfume moulded using curauá fibre/wood flour-based composites for their cosmetic packaging products. The Brazilian branch of Rexam Beauty Packaging, one of the well-known plastic cosmetic packaging products, introduced this product. This new specialty compound meets the customer’s goals of integrating natural components into the packaging as a complement to the all-natural ingredients in its products [112].

### 8.2. Laptop Casing

Presently, laptop casing is made using synthetic polymers such as ABS or polycarbonate. The environmental effect can be reduced by substituting these plastics with natural fibre composites. A hemp fibre plastic composite is one of the potential materials to substitute the synthetic polymers. ABS uses about 100 MJ to make 1 kg of the material, while hemp fibre plastic only uses around 60 MJ of energy to make the same amount of material. This shows that the utilization of energy can be reduced by using natural fibres. The laptop casing made from hemp/PLA based composites would be fully biodegradable and recyclable [113].

### 8.3. Injection-Blow Molded Bottles

National Nutraceutical Center at Clemson University has worked with Plastic Technologies, Inc. (Holland, OH, USA) to produce preforms of PLA and cotton fibres for injection-blow moulded bottles. They are working under a grant for Gaia Herbs, Brevard, to produce an “all-natural” bottle as an alternative to glass bottles. PLA and cotton fibres were compounded using a two-roll mill machine. They included a small amount of herbs such as turmeric in the formulation and found that the bottles have low moisture and oxygen transmission as well as improved UV resistance, 45% higher than that of PET [114].

### 8.4. Biodegradable Food Packaging

The biodegradable plastic food packaging made of 75% PLA and up to 25% wood fibres has been produced by the EU-funded Bio-Based Composite Development project. In the project known as FORBIOPLAST, they are trying to utilize by-products from the forestry and paper-industry to develop biodegradable food packaging. The researchers believe that these products can replace the traditional food tray; however, extensive studies in food packaging are needed. As examples, tests to assess the migration qualities and safety of the material for use with food are essential [115].

### 8.5. Laptop Packaging Made from Blend of Sustainable Plant Fibres

Be Green is a material that produced from plant fibre blend and offers a number of benefits over traditional packaging such as paperboard and plastic. The fibres used are rapidly renewable, abundant and grow like weeds in many parts of the world. Moreover, no petroleum based materials are used in the manufacturing of Be Green’s plant fibre blend [116]. Thus, this material is biodegradable and eco-friendly products.

## 9. Conclusions

The use of natural fibre fabric-biopolymer composites in industrial application provides challenges for researchers around the world to access the performance and quality of natural fibre fabric-biopolymer composites for use as materials in many applications, especially in packaging applications. Natural fibre fabric-biopolymer composites are biodegradable, renewable, and recyclable; they may replace or reduce the use of man-made fibres in various applications. Thus, it is crucial to recognize the functional properties required for natural fibre fabric-biopolymer composites to be used as material for packaging.

Therefore, this review article has focused on the functional properties related to packaging applications of the bamboo fibre fabric-PLA composites. The properties discussed here are required in order to evaluate the feasibility of bamboo fabric-PLA composites in packaging applications. These properties were adapted as per requirement needed by the plastic packaging. Currently, there is no specific standard for the use of natural fibre fabric composites as packaging material. Thus, this review article will help manufacturers and researchers who are interested in studying the essential properties of natural fibre fabric composites for packaging applications. This manuscript will also open new windows and will provide novel insights on the important properties of natural fibre fabric-biopolymer composites, particularly bamboo fabric-PLA composites, for various application-based research.

## Figures and Tables

**Table 1:** Comparison between natural and glass fibres [12].

Natural Fibres	Glass Fibres
Low density	The density is twice of natural fibres
Low cost	It is low cost but higher than natural fibres
Renewability and recyclability	Not renewable and recyclable
Low energy consumption	High energy consumption
Wide distribution	Wide distribution
No abrasion to machines	Abrasion to the machines
No health risk when inhaled	Health risk when inhaled
Biodegradable	Non-biodegradable

**Table 2:** Advantages and disadvantages of natural fibres [7,9,17].

Advantages	Disadvantages
Lower specific weight results in a higher specific strength and stiffness than glass	Lower mechanical properties especially impact resistance
Renewable resource	Heterogeneous quality
Production with low investment at low cost	Moisture sensitivity
Low abrasion, therefore tool wear	Low thermal stability
Non toxic	Low durability
Abundantly available	Poor fire resistance
Biodegradable	Poor fibre-matrix adhesion
Thermal recycling is possible	Price fluctuation by harvest results or agricultural politics

**Table 3:** Physical and mechanical properties of natural fibres and glass fibres [7,18,30,35,36].

Fibre	Length of Fibre (mm)	Diameter of Fibre (µm)	Density (g/cm^3^)	Tensile Strength (MPa)	Young’s Modulus (GPa)	Elongation at Break (%)
Bamboo	2.7	14	0.8–1.1	391–1000	48–89	1.9–3.2
Flax	10–65	5–38	1.4–1.5	800–1500	60–80	1.2–1.6
Hemp	5–55	10–51	1.5	550–900	30–70	1.6
Jute	0.8–6	5–25	1.3–1.46	393–700	10–55	1.5–1.8
Sisal	0.8–8	5–25	1.33–1.45	600–700	22–38	2–3
Ramie	40–250	18–80	1.5	400–938	62–128	2–3.8
Kenaf	1.4–11	12–36	1.2	295	21–60	2.7–6.9
Cotton	15–56	12–35	1.5	287–597	6–12.6	3–10
Banana	0.17	13.16	1.35	529–914	27–33.8	5.3
Pineapple	3–9	20–80	1.5	170–1627	60–83	1–3
Oil palm fibre	0.89–0.99	19.1–25	0.7–1.55	248	3.2	2.5
Bagasse	0.8–2.8	10–34	1.2	20–290	19.7–27.1	3–4.7
E-glass	7	13	2.5	2000–3500	70	2.5

**Table 4:** Chemical components of different fibres [30,32,46].

Chemical Component (%)	Bamboo Fibre	Jute Fibre	Flax Fibre
Aqueous extract	3.16	3.06	5.74
Pectin	0.37	1.72	1.81
Hemicelluloses	12.49	13.53	11.62
Lignin	10.15	13.30	2.78
Cellulose	73.83	68.39	78.05

**Table 5:** Mechanical properties of natural fibre fabric-PLA composites.

Fabric	Fibre Volume Fraction (%)	Fibre Weight Fraction (%)	Tensile Strength (MPa)	Tensile Modulus (GPa)	Flexural Strength (MPa)	Flexural Modulus (MPa)	Reference
Bamboo fabric	-	35	80.64	5.92	143	4495	[23]
Bamboo fabric	51	-	77.58	1.75	-	-	[28]
Flax fabric	-	30	21	1.37	-	-	[60]
Kenaf textiles	-	-	82.28	-	-	-	[61]
Plain weave hemp fabrics (20%)	-	-	64	3.2	-	-	[62]
Twill weaves hemp fabrics (20%)	-	-	70	3.5	-	-	[62]
Denim fabric (3 layers)	-	-	75	4.6	-	-	[5]
Lyocell	-	29.5	60.8	4.48	-	-	[63]
Hemp-untreated	-	30	88.06	10.23	-	-	[64]
Lyocell	-	30	101.23	11.42	-	-	[64]
Lyocell/hemp mixture	-	30	96.01	11.15	-	-	[64]
Manicaria Saccifera	-	-	68.45	4.89	133.12	3.94	[65]

**Table 6:** Chronological order of events in the exploration of natural fibre fabric-biopolymer composites and their related applications.

Year	Natural Fibre Fabric	Biopolymers	Study	Applications	Reference
2000	Jute fabric	Biopol	Study the effects of various chemical surface modifications of jute fabrics as means of improving its suitability as a reinforcement in Biopol based composites	Biocomposites	[103]
2007	Flax fabric	Soy protein resin	Fabrication of environment-friendly ‘green’ composites using modified soy protein concentrate based resins	Biocomposites	[104]
2009	Jute fabric	Poly(butylene succinate) (PBS)	Study the biodegradability of poly(butylene succinate) (PBS)/jute composites	Biocomposites	[105]
2010	Flax non-woven	polyhydroxybutyrate (PHB) and its copolymer with hydroxyvalerate (HV)	Study the influence of addition of flax fibres on the mechanical properties of PHB and PHB/HV copolymer	Biocomposites	[106]
2010	Denim fabric	Poly (lactic) acid (PLA)	To improve the mechanical and thermal properties of PLA resin by using denim woven fabrics	Biocomposites	[5]
2012	Twill and plain weave hemp fabric	Poly (lactic) acid (PLA)	Examine the physical behaviour of hemp/poly(lactic acid) (PLA) composites, particularly the thermal properties and viscoelastic behaviour	Biocomposites	[62]
2012	Bamboo fabric	Poly (lactic) acid (PLA)	Study the development and mechanical characterization of a composite material fabricated from both renewable resources and biodegradable materials: bamboo woven fabric as reinforcement and polylactic acid (PLA) as resin matrix	Biocomposites	[28]
2012	Woven and non-woven jute fabric	Soy resin	Study the mechanical and biodegradation properties of jute, soy matrix and their composites	Biocomposites	[107]
2012	Woven hemp fabric	Polyhydroxybutyrate (PHB)	PHB polymer films and PHB-hemp fabric bio-based composites are subjected to two accelerated weathering procedures	Biocomposites	[108]
2013	Sterculia urens uniaxial fabric	Poly (lactic) acid (PLA)	The effect of fabric surface-treatments on the mechanical and thermal properties of the biocomposites were studied, in order to ascertain whether the PLA and *S. urens* fabric system could effectively be used for making biocomposites suitable for packaging applications	Packaging applications	[109]
2013	Bamboo fabric	Poly (lactic) acid (PLA)	Study the influence of the manufacturing parameters on the mechanical properties of bamboo fabric-PLA prepared via compression moulding method by using Taguchi experimental design approach	Biocomposites	[23]
2014	Bamboo fabric	Poly (lactic) acid (PLA)	To explore the potential of using totally green composites made from renewable resources in packaging applications as compared to conventional thermoplastics	Packaging applications	[69]
2015	Flax fabric	Polyhydroxybutyrate (PHB)	The phenolic contents of flax fibre is studied in more detail in order to determine the impact of the modification on phenylpropanoid metabolism and establish the usefulness of flax products from PHB-overexpressing plants for biomedical applications, particularly wound dressing production	Biomedical applications	[110]
2015	Bark cloth extracted from *F. natalensis*	Biodegradable epoxy resin	Exploratory investigation of *F. natalensis* barks cloth as a reinforcement of new and biodegradable epoxy resin. Develop biodegradable bark cloth reinforced green epoxy composites with view of application to automotive instrument panels	Automotive instrument panel	[111]
2016	Hemp, lyocel fabric	Poly (lactic) acid (PLA)	Compare the mechanical characteristics of uniaxial composites fabricated from reinforcement made from hemp/PLA, hemp–Lyocell/PLA and Lyocell/PLA wrap spun hybrid yarns	Biocomposites	[64]
2016	*Manicaria Saccifera* palm fabric	Poly (lactic) acid (PLA)	Present The development and thermo-mechanical characterization of a novel green composite lamina, made of Poly Lactic Acid (PLA) reinforced with a natural fabric extracted from *Manicaria Saccifera* palm	Biocomposites	[65]

## References

[1] Dicker M.P.M., Duckworth P.F., Baker A.B., Francois G., Hazzard M.K., Weaver P.M. (2014). Green composites: A review of material attributes and complementary applications. Compos. Part A Appl. Sci. Manuf..

[2] La Mantia F.P., Morreale M. (2011). Green composites: A brief review. Compos. Part A Appl. Sci. Manuf..

[3] Abdul Khalil H.P.S., Bhat A.H., Ireana Yusra A.F. (2012). Green composites from sustainable cellulose nanofibrils: A review. Carbohydr. Polym..

[4] Mukherjee T., Kao N. (2011). PLA based biopolymer reinforced with natural fibre: A review. J. Polym. Environ..

[5] Lee J.T., Kim M.W., Song Y.S., Kang T.J., Youn J.R. (2010). Mechanical properties of denim fabric reinforced poly(lactic acid). Fibers Polym..

[6] Hariharan A.B.A., Abdul Khalil H.P.S. (2005). Lignocellulose-based hybrid bilayer laminate composite: Part I—Studies on tensile and impact behavior of oil palm fiber-glass fiber-reinforced epoxy resin. J. Compos. Mater..

[7] Jawaid M., Abdul Khalil H.P.S. (2011). Cellulosic/synthetic fibre reinforced polymer hybrid composites: A review. Carbohydr. Polym..

[8] Faruk O., Bledzki A.K., Fink H.P., Sain M. (2014). Progress report on natural fiber reinforced composites. Macromol. Mater. Eng..

[9] Duhovic M., Peterson S., Jayaraman K., Pickering K. (2008). Natural-fibre-biodegradable polymers composites for packaging. Properties and Performance of Natural-Fibre Composites.

[10] El-Sabbagh A. (2014). Effect of coupling agent on natural fibre in natural fibre/polypropylene composites on mechanical and thermal behaviour. Compos. Part B Eng..

[11] Le Moigne N., Longerey M., Taulemesse J.M., Bénézet J.C., Bergeret A. (2014). Study of the interface in natural fibres reinforced poly(lactic acid) biocomposites modified by optimized organosilane treatments. Ind. Crop. Prod..

[12] Wambua P., Ivens J., Verpoest I. (2003). Natural fibres: Can they replace glass in fibre reinforced plastics?. Compos. Sci. Technol..

[13] Lee S.H., Wang S. (2006). Biodegradable polymers/bamboo fiber biocomposite with bio-based coupling agent. Compos. Part A Appl. Sci. Manuf..

[14] Tajvidi M., Takemura A. (2010). Thermal degradation of natural fiber-reinforced polypropylene composites. J. Thermoplast. Compos. Mater..

[15] Saheb D.N., Jog J.P. (1999). Natural fiber polymer composites: A review. Adv. Polym. Technol..

[16] Oksman K., Wallström L., Berglund L.A., Filho R.D.T. (2002). Morphology and mechanical properties of unidirectional sisal-epoxy composites. J. Appl. Polym. Sci..

[17] Sreekumar P.A., Pickering K.L. (2008). Matrices for natural-fibre reinforced composites. Properties and Performance of Natural-Fibre Composite.

[18] Rowell R.M., Pickering K.L. (2008). Natural fibres: Types and properties. Properties and Performance of Natural-Fibre Composites.

[19] Han G., Lei Y., Wu Q., Kojima Y., Suzuki S. (2008). Bamboo-fiber filled high density polyethylene composites: Effect of coupling treatment and nanoclay. J. Polym. Environ..

[20] Chattopadhyay S.K., Khandal R.K., Uppaluri R., Ghoshal A.K. (2011). Bamboo fiber reinforced polypropylene composites and their mechanical, thermal, and morphological properties. J. Appl. Polym. Sci..

[21] Lobovikov M., Shyam P., Piazza M., Ren H., Wu J. (2007). Non-Wood Forest Products 18 World Bamboo Resources. A Thematic Study Prepared in the Framework of the Global Forest Resources Assessment.

[22] Rassiah K., Megat Ahmad M.M.H., Ali A. (2014). Mechanical properties of laminated bamboo strips from gigantochloa scortechinii/polyester composites. Mater. Des..

[23] Nurul Fazita M.R., Jayaraman K., Bhattacharyya D. (2013). A performance study on composites made from bamboo fabric and poly(lactic acid). J. Reinf. Plast. Compos..

[24] Tokoro R., Vu D.M., Okubo K., Tanaka T., Fujii T., Fujiura T. (2008). How to improve mechanical properties of polylactic acid with bamboo fibers. J. Mater. Sci..

[25] Ma H., Joo C.W. (2011). Influence of surface treatments on structural and mechanical properties of bamboo fiber-reinforced poly(lactic acid) biocomposites. J. Compos. Mater..

[26] Kang J., Kim S. (2011). Improvement in the mechanical properties of polylactide and bamboo fiber biocomposites by fiber surface modification. Macromol. Res..

[27] Okubo K., Fujii T., Thostenson E.T. (2009). Multi-scale hybrid biocomposite: Processing and mechanical characterization of bamboo fiber reinforced PLA with microfibrillated cellulose. Compos. Part A Appl. Sci. Manuf..

[28] Porras A., Maranon A. (2012). Development and characterization of a laminate composite material from polylactic acid (PLA) and woven bamboo fabric. Compos. Part B Eng..

[29] Mohammad Rawi N.F., Jayaraman K., Bhattacharyya D. Optimum processing conditions for the manufacture of bamboo fabric-polypropylene composites. Proceedings of the 11th International Conference Flow Processing in Composite Materials.

[30] Abdul Khalil H.P.S., Bhat I.U.H., Jawaid M., Zaidon A., Hermawan D., Hadi Y.S. (2012). Bamboo fibre reinforced biocomposites: A review. Mater. Des..

[31] Li X. (2004). Physical, Chemical, and Mechanical Properties of Bamboo and Its Utilization Potential for Fiberboard Manufacturing. Ph.D. Thesis.

[32] Yueping W., Ge W., Haitao C., Genlin T., Zheng L., Feng X.Q., Xiangqi Z., Xiaojun H., Xushan G. (2010). Structures of bamboo fiber for textiles. Text. Res. J..

[33] Osorio L., Trujillo E., Van Vuure A.W., Verpoest I. (2011). Morphological aspects and mechanical properties of single bamboo fibers and flexural characterization of bamboo/epoxy composites. J. Reinf. Plast. Compos..

[34] Chen H., Miao M., Ding X. (2009). Influence of moisture absorption on the interfacial strength of bamboo/vinyl ester composites. Compos. Part A.

[35] Olesen P.O., Plackett D.V. Perspective on the performance of natural plant fibres. Proceedings of the Natural Fibres Performance Forum.

[36] Peterson K.S. (2006). Formability and Degradation of Woodfibre-Biopolymer Composite Materials.

[37] Lomov S., Verpoest I., Robitaille F., Long A.C. (2005). Manufacturing and internal geometry of textiles. Design and Manufacture of Textile Composites.

[38] Hearle J.W.S., Chou T.W., Ko F.K. (1989). Mechanics of yarns and nonwoven fabrics. Textile Structural Composites.

[39] Campbell F.C., Campbell F.C. (2010). Thermoplastic composite fabrication processes. Structural Composite Materials.

[40] Shah D.U., Schubel P.J., Clifford M.J. (2013). Modelling the effect of yarn twist of the tensile strength of unidirectional plant fibre yarn composites. J. Compos. Mater..

[41] Kawabata S., Chou T.W., Ko F.K. (1989). Nonlinear mechanics of woven and knitted materials. Textile Structural Composites.

[42] Waite M. (2009). Sustainable textiles: The role of bamboo and a comparison of bamboo textile properties. J. Text. Appar. Technol. Manag..

[43] Liu Y., Hu H. (2008). X-ray diffraction study of bamboo fibers treated with NaOH. Fibers Polym..

[44] Miao M., Finn N. (2008). Conversion of natural fibres into structural composites. J. Text. Eng..

[45] Waite M., Platts J. Engineering Sustainable Textiles: A Bamboo Textile Comparison. http://www.wseas.us/e-library/conferences/2009/vouliagmeni/EELA/EELA-58.pdf.

[46] Li L.J., Wang Y.P., Wang G., Cheng H.T., Han X.J. (2010). Evaluation of properties of natural bamboo fibre for application in summer textiles. J. Fiber Bioeng. Inf..

[47] Fu J., Li X., Gao W., Wang H., Cavaco-Paulo A., Silva C. (2012). Bio-processing of bamboo fibres for textile applications: A mini review. Biocatal. Biotransform..

[48] Patagonia On Bamboo and Rayon. http://www.patagonia.com.au/environment/footprint-chronicles/bamboo-and-rayon/.

[49] Mass E. Bamboo Textiles: Green, Luxurious and Practical. http://www.naturallifemagazine.com/0904/bamboo_textiles.htm.

[50] Ren P., Shen T., Wang F., Wang X., Zhang Z. (2009). Study on biodegradable starch/ommt nanocomposites for packaging applications. J. Polym. Environ..

[51] Guo W., Tao J., Yang C., Song C., Geng W., Li Q., Wang Y., Kong M., Wang S. (2012). Introduction of environmentally degradable parameters to evaluate the biodegradability of biodegradable polymers. PLoS ONE.

[52] Flieger M., Kantorová M., Prell A., Řezanka T., Votruba J. (2003). Biodegradable plastics from renewable sources. Folia Microbiol..

[53] Shen L., Haufe J., Patel M.K. (2009). Product Overview and Market Projection of Emerging Bio-Based Plastics.

[54] Bajpai P.K., Singh I., Madaan J. (2014). Development and characterization of PLA-based green composites: A review. J. Thermoplast. Compos. Mater..

[55] Auras R., Harte B., Selke S. (2004). An overview of polylactides as packaging materials. Macromol. Biosci..

[56] Hamad K., Kaseem M., Deri F. (2011). Effect of recycling on rheological and mechanical properties of poly(lactic acid)/polystyrene polymer blend. J. Mater. Sci..

[57] Smith R. (2005). Biodegradable Polymers for Industrial Applications.

[58] Joffe R., Andersons J., Pickering K. (2008). Mechanical performance of thermoplastic matrix natural-fibre composites. Properties and Performance of Natural-Fibre Composites.

[59] Le Duigou A., Pillin I., Bourmaud A., Davies P., Baley C. (2008). Effect of recycling on mechanical behaviour of biocompostable flax/poly(l-lactide) composites. Compos. Part A Appl. Sci. Manuf..

[60] Kumar R., Yakubu M.K., Anandjiwala R.D. (2010). Biodegradation of flax fiber reinforced poly lactic acid. Express Polym. Lett..

[61] Ben G., Kihara Y. (2007). Development and evaluation of mechanical properties for kenaf fibers/PLA composites. Key Eng. Mater..

[62] Song Y.S., Lee J.T., Ji D.S., Kim M.W., Lee S.H., Youn J.R. (2012). Viscoelastic and thermal behavior of woven hemp fiber reinforced poly(lactic acid) composites. Compos. Part B Eng..

[63] Shibata M., Oyamada S., Kobayashi S., Yaginuma D. (2004). Mechanical properties and biodegradability of green composites based on biodegradable polyesters and lyocell fabric. J. Appl. Polym. Sci..

[64] Baghaei B., Skrifvars M. (2016). Characterisation of polylactic acid biocomposites made from prepregs composed of woven polylactic acid/hemp–lyocell hybrid yarn fabrics. Compos. Part A Appl. Sci. Manuf..

[65] Porras A., Maranon A., Ashcroft I.A. (2016). Thermo-mechanical characterization of manicaria saccifera natural fabric reinforced poly-lactic acid composite lamina. Compos. Part A Appl. Sci. Manuf..

[66] Brown M. Meeting Sustainability Goals Using Thermoformed Packaging. http://packaging2.com/PDF/SPF%20text%20a%2093005.pdf.

[67] Silvestre C., Duraccio D., Cimmino S. (2011). Food packaging based on polymer nanomaterials. Prog. Polym. Sci..

[68] Katiyar V., Gaur S.S., Pal A.K., Kumar A., Alavi S., Thomas S., Sandeep K.P., Kalarikkal N., Varghese J., Yaragalla S. (2015). Properties of plastics for packaging applications. Polymers for Packaging Applications.

[69] Nurul Fazita M.R., Jayaraman K., Bhattacharyya D. (2014). Bamboo fabric reinforced polypropylene and poly(lactic acid) for packaging applications: Impact, thermal, and physical properties. Polym. Compos..

[70] Bax B., Müssig J. (2008). Impact and tensile properties of PLA/Cordenka and PLA/flax composites. Compos. Sci. Technol..

[71] Bledzki A.K., Jaszkiewicz A. (2010). Mechanical performance of biocomposites based on PLA and PHBV reinforced with natural fibres—A comparative study to PP. Compos. Sci. Technol..

[72] Bledzki A.K., Jaszkiewicz A., Scherzer D. (2009). Mechanical properties of PLA composites with man-made cellulose and abaca fibres. Compos. Part A Appl. Sci. Manuf..

[73] Jandas P.J., Mohanty S., Nayak S.K., Srivastava H. (2011). Effect of surface treatments of banana fiber on mechanical, thermal, and biodegradability properties of PLA/banana fiber biocomposites. Polym. Compos..

[74] Liang J.Z., Li R.K.Y., Tjong S.C. (2000). Effects of filler content and size on drop-weight dart impact fracture behavior of glass bead-filled polypropylene composites. J. Thermoplast. Compos. Mater..

[75] (2007). ASTM D7136-07. Standard Test Method for Measuring the Damage Resistance of a Fiber-Reinforced Polymer Matrix Composites to a Drop Weight Impact Event.

[76] Wambua P., Hu H., Verpoest I. (2007). Impact response of hemp/polypropylene based composites. Kenya J. Mech. Eng..

[77] Bledzki A.K., Faruk O. (2004). Creep and impact properties of wood fibre–polypropylene composites: Influence of temperature and moisture content. Compos. Sci. Technol..

[78] Bledzki A.K., Faruk O. (2003). Wood fibre reinforced polypropylene composites: Effect of fibre geometry and coupling agent on physico-mechanical properties. Appl. Compos. Mater..

[79] Auras R.A., Singh S.P., Singh J.J. (2005). Evaluation of oriented poly(lactide) polymers *vs.* Existing PET and oriented PS for fresh food service containers. Pack. Technol. Sci..

[80] Lee C.H., Lou C.W., Hsing W.H., Tsai I.J., Lin J.H. (2008). Thermoplastic polyurethane (TPU) honeycomb air cushion combined with polylactic acid (PLA) nonwoven fabric for impact protection. Adv. Mater. Res..

[81] Lou C.W., Lu C.T., Lin C.M., Lee C.H., Chao C.Y., Lin J.H. (2010). Process technology and performance evaluation of functional knee pad. Fibers Polym..

[82] Shi Q.F., Mou H.Y., Li Q.Y., Wang J.K., Guo W.H. (2012). Influence of heat treatment on the heat distortion temperature of poly(lactic acid)/bamboo fiber/talc hybrid biocomposites. J. Appl. Polym. Sci..

[83] Best in Packaging Bio-Plastics: From Hot to Cold. http://bestinpackaging.com/2010/09/12/bio-plastics-from-hot-to-cold/.

[84] Plastic Ingenuity Plastic ingenuity Increases PLA Heat Deflection Temperature. http://www.plasticingenuity.com/blog/recent-news/plastic-ingenuity-increases-pla-heat-deflection-temperature/.

[85] Huda M.S., Drzal L.T., Misra M., Mohanty A.K., Williams K., Mielewski D.F. (2005). A study on biocomposites from recycled newspaper fiber and poly(lactic acid). Ind. Eng. Chem. Res..

[86] Baley C., Boulmard A. Recycling Composite Materials Reinforced with Plant Fibres. http://www.jeccomposites.com/news/composites-news/recycling-composite-materials-reinforced-plant-fibres#.TzmUy9Telag.email.

[87] Pillin I., Montrelay N., Bourmaud A., Grohens Y. (2008). Effect of thermo-mechanical cycles on the physico-chemical properties of poly(lactic acid). Polym. Degrad. Stab..

[88] Bourmaud A., Baley C. (2007). Investigations on the recycling of hemp and sisal fibre reinforced polypropylene composites. Polym. Degrad. Stab..

[89] Gassan J., Bledzki A.K. (2001). Thermal degradation of flax and jute fibers. J. Appl. Polym. Sci..

[90] Nurul Fazita M.R., Krishnan J., Debes B., Sohrab H.M.K., Mohamad H., Abdul K.H.P.S. (2015). Disposal options of bamboo fabric-reinforced poly(lactic) acid composites for sustainable packaging: Biodegradability and recyclability. Polymers.

[91] Tokiwa Y., Calabia B. (2006). Biodegradability and biodegradation of poly(lactide). Appl. Microbiol. Biotechnol..

[92] Ochi S. (2008). Mechanical properties of kenaf fibers and kenaf/PLA composites. Mech. Mater..

[93] Mathew A.P., Oksman K., Sain M. (2005). Mechanical properties of biodegradable composites from poly lactic acid (PLA) and microcrystalline cellulose (MCC). J. Appl. Polym. Sci..

[94] Yussuf A., Massoumi I., Hassan A. (2010). Comparison of polylactic acid/kenaf and polylactic acid/rise husk composites: The influence of the natural fibers on the mechanical, thermal and biodegradability properties. J. Polym. Environ..

[95] Rudeekit Y., Numnoi J., Tajan M., Chaiwutthinan P., Leejarkpai T. (2008). Determining biodegradability of polylactic acid under different environments. J. Met. Mater. Miner..

[96] Kolstad J.J., Vink E.T.H., De Wilde B., Debeer L. (2012). Asessment of anaerobic degradation of ingeo polylactides under accelerated landfill conditions. Polym. Degrad. Stab..

[97] Shi B., Palfery D. (2012). Temperature-dependent polylactic acid (PLA) anaerobic biodegradablity. Int. J. Environ. Waste Manag..

[98] Yu T.X., Tao X.M., Xue P. (2000). The energy-absorbing capacity of grid-domed textile composites. Compos. Sci. Technol..

[99] Xue P., Yu T.X., Tao X.M. (2000). Effect of cell geometry on the energy-absorbing capacity of grid-domed textile composites. Compos. Part A Appl. Sci. Manuf..

[100] Jacob G.C., Fellers J.F., Simunovic S., Starbuck J.M. (2002). Energy absorption in polymer composites for automotive crashworthiness. J. Compos. Mater..

[101] Mamalis A.G., Robinson N.M., Manolakos D.E., Demosthenous G.A., Ioannidis M.B., Carruthers J. (1997). Crashworthy capability of composite material structures. Compos. Struct..

[102] Gupta N.K., Easwara Prasad G.L. (1999). Quasi-static and dynamic axial compression of glass/polyester composite hemi-spherical shells. Int. J. Impact Eng..

[103] Mohanty A.K., Khan M.A., Hinrichsen G. (2000). Surface modification of jute and its influence on performance of biodegradable jute-fabric/biopol composites. Compos. Sci. Technol..

[104] Huang X., Netravali A. (2007). Characterization of flax fiber reinforced soy protein resin based green composites modified with nano-clay particles. Compos. Sci. Technol..

[105] Liu L., Yu J., Cheng L., Yang X. (2009). Biodegradability of poly(butylene succinate) (PBS) composite reinforced with jute fibre. Polym. Degrad. Stab..

[106] Barkoula N.M., Garkhail S.K., Peijs T. (2010). Biodegradable composites based on flax/polyhydroxybutyrate and its copolymer with hydroxyvalerate. Ind. Crops Prod..

[107] Behera A.K., Avancha S., Basak R.K., Sen R., Adhikari B. (2012). Fabrication and characterizations of biodegradable jute reinforced soy based green composites. Carbohydr. Polym..

[108] Michel A.T., Billington S.L. (2012). Characterization of poly-hydroxybutyrate films and hemp fiber reinforced composites exposed to accelerated weathering. Polym. Degrad. Stab..

[109] Jayaramudu J., Reddy G.S.M., Varaprasad K., Sadiku E.R., Ray S.S., Rajulu A.V. (2013). Structure and properties of poly (lactic acid)/sterculia urens uniaxial fabric biocomposites. Carbohydr. Polym..

[110] Kulma A., Skórkowska-Telichowska K., Kostyn K., Szatkowski M., Skała J., Drulis-Kawa Z., Preisner M., Żuk M., Szperlik J., Wang Y.F. (2015). New flax producing bioplastic fibers for medical purposes. Ind. Crop. Prod..

[111] Rwawiire S., Tomkova B., Militky J., Jabbar A.K., Bandu M. (2015). Development of a biocomposite based on green epoxy polymer and natural cellulose fabric (bark cloth) for automotive instrument panel applications. Compos. Part B Eng..

[112] SABIC Innovative Plastics Sabic Innovative Plastics’ New Bio-Based Composites Give Customers Greatly Expanded Options for More Impactful Sustainable Products. http://www.pressreleasefinder.com/pr/SABICIPPR093/en.

[113] Davies J. Jack Davies Design. http://cargocollective.com/jackdaviesdesign/Sustainable-laptop-casing.

[114] Joseph A.G. Biopolymers Strive to Meet Price/Performance Challenge. http://www.ptonline.com/articles/biopolymers-strive-to-meet-price-performance-challenge.

[115] Anton S. Developments in Wood-Fibre Based Packaging Material. https://bestinpackaging.com/2012/02/28/developments-in-wood-fibre-based-packaging-material/.

[116] Be Green Packaging LLC Google Chromebook Goes Green: New Laptop Packaging Made from Blend of Sustainable Plant Fibers. https://begreenpackaging.wordpress.com/2013/10/28/google-chromebook-be-green-packaging/.

